# Prenatal DDT Exposure in Relation to Anthropometric and Pubertal Measures in Adolescent Males

**DOI:** 10.1289/ehp.7287

**Published:** 2004-09-07

**Authors:** Beth C. Gladen, Mark A. Klebanoff, Mary L. Hediger, Solomon H. Katz, Dana B. Barr, Mark D. Davis, Matthew P. Longnecker

**Affiliations:** ^1^National Institute of Environmental Health Sciences, National Institutes of Health, Department of Health and Human Services, Research Triangle Park, North Carolina, USA; ^2^National Institute of Child Health and Human Development, National Institutes of Health, Department of Health and Human Services, Bethesda, Maryland, USA; ^3^Krogman Center for Research in Child Growth and Development, University of Pennsylvania, Philadelphia, Pennsylvania, USA; ^4^National Center for Environmental Health, Centers for Disease Control and Prevention, Department of Health and Human Services, Atlanta, Georgia, USA

**Keywords:** child development, DDE, DDT, growth, prenatal exposure delayed effects, puberty

## Abstract

DDT (dichlorodiphenyltrichloroethane), a pesticide once used widely in agriculture and now limited to public health use, remains a controversial chemical because of a combination of benefits and risks. DDT or its breakdown products are ubiquitous in the environment and in humans. Compounds in the DDT family have endocrine actions and have been associated with reproductive toxicity. A previous study reported associations between prenatal exposure to *p,p*′-DDE [1,1-dichloro-2,2-bis(*p*-chlorophenyl)-ethylene] and increased height and weight in adolescent boys. We examined a group with higher exposures to see whether similar associations would occur. Our study group was 304 males born in Philadelphia in the early 1960s who had participated in a previous study. Anthropometric and pubertal measures from one to six visits during their adolescent years were available, as were stored maternal serum samples from pregnancy. We measured *p,p*′-DDE, *p,p*′-DDT [1,1,1-trichloro-2,2-bis(*p*-chlorophenyl)-ethane], and *o,p*′-DDT [1,1,1-trichloro-2-(*o*-chlorophenyl)-2-(*p*-chlorophenyl)-ethane] in the maternal serum. Outcomes examined in the boys were height, ratio of sitting height to height, body mass index, triceps skinfold thickness, ratio of subscapular to the sum of triceps and subscapular skinfold thicknesses, skeletal age, serum testosterone, and serum dehydroepiandrosterone sulfate. No associations between prenatal exposure to any of the DDT compounds and any outcome measure were seen.

The combination of public health benefits and environmental risks associated with DDT has made use of the pesticide controversial (Walker et al. 2003). DDT was once used extensively throughout the world, for both agricultural and public health purposes [Agency for Toxic Substances and Disease Registry (ATSDR) 2002]. Use today is generally limited to vector control, primarily of malaria, and is covered as of May 2004 by the Stockholm Convention on Persistent Organic Pollutants, a treaty signed by 151 countries and currently ratified by more than 75 countries (Stockholm Convention on Persistent Organic Pollutants 2004). Decisions in any specific instance about whether to use DDT even for vector control can be hotly debated (Wendo 2004).

Use of DDT in the United States began in the 1940s, peaked in the early 1960s, and essentially ended in 1972. Timelines in other countries differed but generally followed the same pattern of steep rise and fall in amounts used. Despite the decline in use, several components and breakdown products of the pesticide are still widely detectable in the environment and in humans (Jaga and Dharmani 2003; Smith 1999). The pesticide product consists primarily of the actual insecticide *p,p*′-DDT [1,1,1-trichloro-2,2-bis(*p*-chlorophenyl)-ethane], with *o,p*′-DDT [1,1,1-trichloro-2-(*o*-chlorophenyl)-2-(*p*-chlorophenyl)-ethane] and several other minor components making up the remainder (ATSDR 2002). The primary degradation product and human metabolite of *p,p*′-DDT is *p,p*′-DDE [1,1-dichloro-2,2-bis(*p*-chlorophenyl)-ethylene]; the latter is also the most persistent member of the DDT family and the one that bioaccumulates most extensively in humans.

The various components of the DDT family have a number of known biologic actions. The insecticidal effects of *p,p*′-DDT are attributable to neurotoxicity. A number of endocrine effects have been shown, including antiandrogenic properties of *p,p*′-DDE (Gray et al. 2001), estrogenic properties of *o,p*′-DDT (Kupfer 1975), and modulation of steroid hormone homeostasis through induction of hepatic enzymes (Wyde et al. 2003; You et al. 2001). Changes in immune markers have been seen (Vine et al. 2001). Carcinogenicity has been demonstrated in animals, although evidence in humans is mixed (Turusov et al. 2002). Associations with impaired lactation have been reported (Gladen and Rogan 1995; Rogan et al. 1987). Reproductive effects have been shown as well. A large human study has shown associations of maternal *p,p*′-DDE with preterm birth and decreased birth weight (Longnecker et al. 2001). Animal studies with *p,p*′-DDE show a number of reproductive abnormalities in male offspring (Gray et al. 2001); whether similar effects are seen in humans at usual exposure levels is uncertain (Flores-Luévano et al. 2003; Hosie et al. 2000; Longnecker et al. 2002). Paternal occupational DDT exposure has also been associated with birth defects (Salazar-García et al. 2004).

Prenatal exposure to DDT can also have delayed effects. There are reports of neurotoxic and immunotoxic effects in young children (Dewailly et al. 2000; Ribas-Fitó et al. 2003), although not all studies show such effects (Gladen et al. 1988; Rogan et al. 1987). At even later ages, prenatal and lactational exposure to *p,p*′-DDE in animals has been associated with delayed male puberty in some but not all studies (Loeffler and Peterson 1999; You et al. 1998).

A previous human study showed that adolescent males with higher prenatal exposure to *p,p*′-DDE had increases in both height and body mass index (BMI) compared with those with lower exposures; markers of puberty were unaffected (Gladen et al. 2000). In view of the continuing controversy about DDT and concerns about substantial increases in childhood obesity (Ogden et al. 2002), we examined another population with higher exposures to see whether similar effects in adolescent males would be seen there.

## Materials and Methods

### Subjects.

The Collaborative Perinatal Project (CPP) was a large multicenter prospective study of approximately 50,000 children born between 1959 and 1966 (Broman 1984; Hardy 2003). Data collected included background information on the mothers obtained from questionnaires during pregnancy. Serum samples taken from the mothers during pregnancy were stored, and many are still available.

The Philadelphia Blood Pressure Project (PBPP) followed some of the children enrolled at one of the CPP centers during adolescence and early adulthood (Katz et al. 1980). This was an urban population; the children had been born at Pennsylvania Hospital and followed at Children’s Hospital of Philadelphia. Several subgroups were chosen for study; one was a stratified random sample of those enrolled in the CPP who were born between 1961 and 1965. These subjects were seen annually up to three times in 1977–1980 and again annually up to another three times in 1982–1985. Data collected included anthropometric measurements and pubertal markers.

The subjects of the present study were chosen from among the 373 singleton males from the random sample studied in the PBPP. Of those, 314 had stored maternal samples from the third trimester available. Those samples were shipped to the Centers for Disease Control and Prevention and analyzed for several DDT compounds. Of the samples shipped, nine had insufficient quantity for analysis, one was lost during analysis, and 304 were successfully analyzed. The 304 boys whose maternal samples were analyzed are the subjects of this report.

### Primary variables.

Anthropometric measurements up to 20 years of age from the PBPP were used; data from a total of 1,137 visits from the 304 boys were available. Measurement techniques and reliabilities have been discussed previously (Katz et al. 1980; Tanner et al. 1969). Quantities measured at all visits included height, sitting height, weight, triceps skinfold thickness, and subscapular skinfold thickness. Height and sitting height were measured using a Holtain stadiometer (Holtain Ltd., Crymych, Wales). Weight was measured on a Health-O-Meter beam balance scale (Health-O-Meter, Bridgeview, IL). Skinfolds were measured using a Holtain skinfold caliper. We examined several measures of overall size and body proportion: height, the ratio of sitting height to height (height ratio), BMI, triceps skinfold thickness, and the ratio of subscapular to the sum of sub-scapular and triceps skinfold thicknesses (a measure of central adiposity). Missing data were minimal: Height was unavailable four times, sitting height 14 times, weight seven times, triceps skinfold thickness four times, and sub-scapular skinfold thickness seven times.

Skeletal age was determined at the three PBPP visits in 1977–1980, using the Tanner-Whitehouse II method of rating hand–wrist radiographs on the maturity of 20 individual bones (Katz et al. 1980; Tanner et al. 1975). Skeletal age was unavailable for 4% of the visits where it was scheduled to be done.

Testosterone was measured at the first two PBPP visits in 1977–1979 by radio-immunoassay on samples of venous blood collected at the time of examination (Furuyama et al. 1970; Zemel and Katz 1986). Dehydroepiandrosterone sulfate (DHEAS) was measured only at the second visit in 1978–1979, again by radioimmunoassay (Buster and Abraham 1972). Not all boys volunteered for the blood draw; testosterone was unavailable for 23% of the visits where it was scheduled to be done, and DHEAS was unavailable in 22%.

Maternal serum samples were analyzed for *p,p*′-DDE, *p,p*′-DDT, and *o,p*′-DDT using a semiautomated solid-phase extraction and gel permeation chromatography cleanup followed by an isotope dilution gas chromatography–high resolution mass spectrometry analysis (Barr et al. 2003; Sandau et al. 2003). Recovery correction was done for each analyte in each individual sample. For 25 samples, *p,p*′-DDT could not be measured because of quality control limit failure. Cholesterol and triglycerides were also measured using standard clinical assays. Total serum lipids were calculated as 62.3 + 2.27 cholesterol + triglycerides (Phillips et al. 1989). Pesticide concentrations were reported as nanograms of pesticide per gram total serum lipids. The sum of the three DDT compounds was calculated, unless *p,p*′-DDT could not be measured. Samples with nondetectable amounts for *o,p*′-DDT or *p,p*′-DDT were considered to be zero; because detection limits were low, imputing any other value up to the detection limit changed the sum of DDT by < 1% and never changed the categories when exposure was categorized.

### Statistical analysis.

We used models to examine outcome measurements in relation to pesticide concentration after adjustment for important predictors and potential confounders. The age of the boy at examination is a key predictor and was included as a cubic polynomial to allow for nonlinearity. Parental size is also a strong predictor of child size; maternal height and prepregnancy BMI were available and were included as linear terms, but paternal size was not available. We also adjusted for breast-feeding (yes, no), maternal smoking at the time of pregnancy (yes, no), number of older siblings (0, 1, ≥ 2), race (African American, white), maternal age at birth (13–19, 20–24, 25–29, ≥ 30 years), and maternal age at menarche (8–11, 12, 13, ≥14 years). We used the family socioeconomic index (SEI) score, based on education, occupation, and income, created by the CPP investigators for internal comparisons (Myrianthopoulos and French 1968). Median SEI for the entire CPP was 4.3, with a range from 0 to 9.5; scores here were categorized into three groups (0–2.5, 2.6–5.0, ≥5.1). A term for each boy was included as a random effect to account for the correlation among multiple measures of the same boy. For triceps skinfold thickness and testosterone, the analysis was done on a log scale. Tests of statistical significance reported are either tests of whether categories differ for discrete predictors or tests for zero slope for continuous predictors. Models were fit using SAS version 9 (SAS Institute Inc., Cary, NC).

## Results

Most of the 304 boys in this study were African American (Table 1). Most of their mothers reported smoking during pregnancy, and few mothers breast-fed their sons. One-quarter of the boys were first-born. The distribution of family SEI was similar to that of all African Americans in the CPP. Other characteristics of the mother and family around the time of birth are shown in Table 1.

Concentrations of *p,p*′-DDE in maternal serum during pregnancy ranged from 1 to 25 μg/g lipid (Table 2), with a median of 5.7 μg/g lipid. The other two DDT compounds were present at lower concentrations; median *p,p*′-DDT was 1.9 μg/g lipid, and median *o,p*′-DDT was 0.14 μg/g lipid. The three compounds measured were correlated; the correlation of *p,p*′-DDE with *p,p*′-DDT was 0.65 and with *o,p*′-DDT was 0.58, whereas *p,p*′-DDT and *o,p*′-DDT had a correlation of 0.77.

The boys had from one to six adolescent visits with anthropometric measurements available; 20% had one or two, 34% had three, and 47% had four or more. Age at the first measurement ranged from 10.8 to 17.9 years, with a median of 12.8 years. Age at the last measurement ranged from 12.2 to 20.0 years, with a median of 17.6 years. Age at first measurement was a major determinant of number of measurements, because those who were older at the start of follow-up left the age range of interest more quickly. In addition, no whites had more than three visits.

Height ranged from 132 to 196 cm, with the expected strong relationship to age. Selected percentiles at each age are shown in Table 3. Height ratio ranged from 46 to 55%. BMI was skewed, ranging from 14 to 45 kg/m^2^. Triceps skinfold was more skewed, ranging from 3.5 to 43.4 mm. Central adiposity ranged from 31 to 78%.

The boys also had up to three measures of skeletal age; 66% had all three measurements, and two boys had none. Skeletal age ranged from 8.7 to 18 years. In addition, 260 boys had one or two testosterone measurements available, and 213 had a single measurement of DHEAS. Testosterone ranged from 1 to 1,258 ng/dL; DHEAS ranged from 56 to 5,600 ng/mL.

The crude relationships of these anthropometric and pubertal measures to maternal prenatal concentrations of *p,p*′-DDE are shown in Table 4 for several age ranges. Not all boys had measurements available in all age ranges; for skeletal ages and hormones, there were few measurements past 17 years of age. Little systematic relationship to *p,p*′-DDE was seen for any of the measures.

Models as described above were fit to these measures to allow age to be treated as a continuous predictor and to adjust for other predictors and potential confounders (Table 5). In no case was *p,p*′-DDE a statistically significant predictor of the outcome (all *p* > 0.10). Effects of other predictors were seen. All outcomes were significantly related to the age of the boy at measurement (data not shown). Height also increased with maternal height (*p* < 0.001) and maternal BMI (*p* = 0.053); first-born boys also had higher means (*p* = 0.043), as did those from families with higher SEI (*p* = 0.094). Height ratio decreased with maternal height (*p* < 0.001); whites also had larger means (*p* < 0.001), as did later-born children (*p* = 0.063) and those whose mothers had early menarche (*p* = 0.095). BMI increased with maternal BMI (*p* < 0.001). Triceps skinfold increased with maternal BMI (*p* < 0.001); first-born boys (*p* = 0.037) and whites (*p* = 0.056) had higher means. Mean central adiposity was higher in African Americans (*p* = 0.002). Testosterone was increased among those whose mothers had early menarche (*p* = 0.052) and those from families with higher SEI (*p* = 0.092). Skeletal age and DHEAS showed no significant effects of predictors other than age of the boy.

Use of *p,p*′-DDT, *o,p*′-DDT, or the sum of the three compounds rather than *p,p*′-DDE as the exposure also resulted in no significant effects on any of the outcomes analyzed (data not shown). When the analysis shown in Table 5 was done separately for each of the age groups used in Table 4, the results were again not significant with one exception. At the youngest ages, the five exposure groups had significantly different BMIs, but the pattern was not monotonic in dose; as with the crude results in Table 4, the highest BMIs were seen for the 3–6 μg/g dose group. If the analysis shown in Table 5 is restricted to African Americans, the results are essentially unchanged (data not shown).

## Discussion

In this study, we found no association of pre-natal exposure to *p,p*′-DDE, *p,p*′-DDT, or *o,p*′-DDT with any of the anthropometric or pubertal measures we examined in adolescent males. In a previous study of 278 adolescent boys and 316 girls, prenatal *p,p*′-DDE exposure was also not related to pubertal markers (Gladen et al. 2000). However, increased exposure in that study was associated with greater height and BMI of the boys. The subjects of the present study, who were born during the peak of DDT use in the United States, had higher exposures than those in the previous study, who were born after agricultural DDT use had been banned. Median *p,p*′-DDE in maternal serum in the present study was 5.7 μg/g serum lipid; the median in the previous study was approximately equivalent to 1.6 μg/g serum lipid [12.6 ng/g serum (Rogan et al. 1986), converted assuming 8 g lipid/L serum (Longnecker et al. 2003)]. The failure to replicate the previous findings on height and BMI in the present study with higher exposures raises the possibility that the earlier results may have been due to chance. However, there were a number of differences in the populations studied; for example, the previous study subjects were mostly whites, were mostly breast-fed, and had mothers who were of higher socioeconomic status and less likely to smoke.

Childhood concentration of *p,p*′-DDE has also been studied in relation to childhood height and pubertal development (Denham et al. 2004; Karmaus et al. 2002), and adult concentration of *p,p*′-DDE has been studied in relation to testosterone and DHEAS (Ayotte et al. 2001; Hagmar et al. 2001; Martin et al. 2002; Persky et al. 2001). However, these studies have limited relevance to the question addressed here. Prenatal exposure is likely to act through different mechanisms than does postnatal exposure. Childhood concentrations of persistent organochlorines such as the DDT compounds are poor surrogates for prenatal exposures, because concentrations even into adolescence are most strongly determined by breast-feeding (Jacobson et al. 1989; Karmaus et al. 2001; Nawrot et al. 2002).

The adolescent period studied here is a time of rapid development, with changes in body size and proportions, development of secondary sexual characteristics, skeletal maturation, and changes in the hormonal milieu all occurring. Prenatal exposure to compounds with endocrine activity might influence either the timing or the ultimate result of any or all of these changes. All of the outcome measures we studied reflect some aspect of adolescent development, although we do lack some classic outcomes such as Tanner stages and time of peak height velocity. Height increases with age, although it levels off in the later teens; height ratio first declines and then increases with age as body proportions shift (Hamill et al. 1973; Malina et al. 1974). BMI increases with age, albeit with considerable variability. Triceps skinfold thickness declines with age, again with considerable variability; it also becomes smaller relative to subscapular skin-fold thickness, such that central adiposity increases. Skeletal age increases with chronological age, up to full maturity at skeletal age 18. Testosterone and DHEAS concentrations increase with age, with considerable variability. The substantial variability seen with some of these measures means that our failure to find associations of prenatal exposure to the DDT compounds with any of these outcomes could be due to inadequate power, although the patterns of the observed relationships do not suggest this explanation.

We did have adequate power to discern effects of other known predictors. As expected, height and BMI of boys were influenced by the height and BMI of their mothers, consistent with previous work (Celi et al. 2003; Wingerd and Schoen 1974). The racial differences we saw were consistent with those seen elsewhere. In a national survey, the relationship of height and weight to race was inconsistent across age, but height ratio showed a clear racial difference (Hamill et al. 1973). In the same survey, whites had greater triceps skinfolds than did African Americans but similar subscapular skinfolds, leading to lower central adiposity (Johnston et al. 1974); racial differences in skeletal age were inconsistent across ages (Roche et al. 1975, 1978). First-born children have been shown to be taller and heavier (Celi et al. 2003; Ong et al. 2002; Wingerd and Schoen 1974), consistent with our findings; increased skinfold thickness among first-borns has also been reported in another investigation based on the PBPP (Stettler et al. 2000). Prenatal exposure to smoking has been associated with decreased height (Fogelman 1980) and increased obesity (Power and Jefferis 2002); our results were in the expected direction, although they did not achieve statistical significance. Our sample included very few breast-fed children, consistent with the low overall breast-feeding rates at that time and with the lower rates in African Americans and in the Northeast (Hirschman and Hendershot 1979), so the failure to see any associations of our outcomes to breast-feeding was not surprising.

Although there are many early influences on later development, such as the association of prenatal exposure to certain antipsychotic drugs with later height (Platt et al. 1988), most would be expected to be unrelated to exposure to DDT and thus are not candidate confounders. We controlled for the most likely confounders, although observational studies are always subject to potential residual confounding. We had no information about maternal diet before pregnancy or about the diet of the child after birth; maternal diet is a predictor of the exposure, and childhood diet is a predictor of growth and development. Adjusting for paternal size might have made our estimates more precise, but this information was not available. Birth weight has been shown to be related to prenatal *p,p*′-DDE exposure in the CPP population (Longnecker et al. 2001); however, we did not consider it appropriate to adjust for birth weight because this is an intermediate variable in the relationships between exposure and adolescent outcomes.

We measured exposure using third-trimester serum samples. Specific aspects of prenatal development occur during critical windows, so timing of exposure can be important. However, for persistent compounds such as DDT, concentrations are generally stable over periods of months or longer, with little variation over the course of pregnancy (Longnecker et al. 1999). The analytical methods used were sensitive, selective, and reliable, with relative standard deviations, including both the error from the sample preparation and the instrumental methods, of 11% and detection limits in the low picograms per millilliter range (Barr et al. 2003).

The participants in the study were not a random sample of the general population. Those enrolled in the CPP from the Philadelphia study center were clinic patients who were planning to deliver at the study hospital (Broman 1984); they were mostly African American and relatively low income, representative of the population obtaining medical care at this clinic. The PBPP study group was a random sample of the CPP study group (Katz et al. 1980). We have no information about exposure among those who chose not to participate in either the base CPP study or the PBPP follow-up, but there is no reason to anticipate that prenatal DDT exposure would differ between participants and nonparticipants. Refusal rates were greatest for the hormone measurements, but there was little systematic relationship to exposure; for example, among African Americans, those with both testosterone measurements available had a median *p,p*′-DDE of 6.2, whereas those with none or one, due to either a missed visit or a refusal, had a median of 6.3.

In summary, we have seen no association between prenatal exposure to DDT-related compounds and several anthropometric and pubertal measures in males. However, high variability in some of the outcome measures means we cannot rule out subtle changes.

## Figures and Tables

**Table 1:** Background characteristics of boys and their families.

Characteristic	Percent
Race	
White	15
African American	85
Maternal smoking at time of pregnancy	
No	44
Yes	56
Breast-fed	
Yes	6
No	94
No. of older siblings	
0	25
1	25
≥ 2	50
Maternal height (cm)	
144–155.9	29
156–165.9	54
166–181	17
Maternal prepregnancy BMI (kg/m^2^)	
16–19.9	21
20–24.9	47
25–29.9	23
30–44	9
Maternal age at menarche (years)	
8–11	21
12	29
13	25
≥ 14	26
Maternal age at enrollment in CPP (years)	
13–19	23
20–24	36
25–29	23
30–42	18
Family SEI at time of pregnancy	
0–2.5	17
2.6–5.0	59
≥ 5.1	24

Number of cases (of 304) with missing data: three for breast-fed, one for number of older siblings, four for maternal height, six for maternal prepregnancy BMI, two for maternal age at menarche, 10 for family SEI at time of pregnancy.

**Table 2:** Distribution of chemical concentrations in maternal serum.

Chemical	Concentration (μg/g lipid)	Percent
*p,p*′-DDE	1.0–2.9	14
	3.0–5.9	38
	6.0–8.9	24
	9.0–11.9	13
	12.0–25.1	11
*p,p*′-DDT*^a^*	ND*^b^*–0.9	17
	1.0–1.9	36
	2.0–2.9	24
	3.0–3.9	11
	4.0–12.7	11
*o,p*′-DDT	ND*^c^*–0.07	29
	0.08–0.15	28
	0.16–0.23	18
	0.24–0.31	10
	0.32–1.33	14
∑ DDT*^a^*	1.8–3.9	11
	4.0–7.9	37
	8.0–11.9	28
	12.0–15.9	13
	16.0–33.1	11

ND, not detected.

a Not available for 25 (of 304) boys because of quality control limit failure.

b One sample had nondetectable *p*,*p*′-DDT (detection limit, 0.01 μg/g lipid).

c Fifteen samples had non-detectable *o,p*′-DDT (detection limits, 0.007–0.054 μg/g lipid).

**Table 3:** Percentiles of anthropometric and pubertal measures by age range (years).

Measure	10–10.9	11–11.9	12–12.9	13–13.9	14–14.9	15–15.9	16–16.9	17–17.9	18–18.9	19–20.0
Height (cm)										
No.	10	97	149	185	140	111	105	118	111	107
90th	—	157	165	171	177	180	184	185	185	185
Median	140	145	151	158	166	171	173	173	175	176
10th	—	138	143	147	154	158	166	167	168	167
Height ratio (%)										
No.	10	97	148	185	140	110	103	117	108	105
90th	—	52.9	52.5	52.3	52.2	53.0	52.6	52.7	52.4	52.3
Median	52.0	51.1	50.5	50.3	50.1	50.2	50.5	50.8	50.8	50.8
10th	—	49.4	49.0	48.8	48.7	48.3	48.4	49.0	49.1	49.4
BMI (kg/m^2^)										
No.	10	97	149	185	139	111	105	118	110	106
90th	—	22.0	23.9	23.7	24.2	25.2	24.6	27.5	27.5	27.8
Median	17.2	17.4	18.0	18.6	19.3	20.4	20.4	21.4	22.2	21.8
10th	—	15.2	15.4	15.9	16.1	17.6	17.9	19.0	19.2	19.1
Triceps skinfold thickness (mm)										
No.	10	97	149	185	140	111	105	118	111	107
90th	—	16.9	18.2	16.7	16.1	17.1	10.3	15.0	15.3	14.6
Median	7.8	8.1	7.8	7.4	7.1	7.0	6.6	6.8	7.3	6.9
10th	—	5.6	5.2	4.9	5.0	5.2	4.6	5.0	4.9	4.5
Central adiposity (%)										
No.	9	97	148	185	140	110	105	118	111	107
90th	—	52	54	56	56	58	60	63	64	65
median	44	46	47	48	49	52	55	56	56	58
10th	—	40	41	41	42	42	46	49	49	50
Skeletal age (years)										
No.	9	95	146	177	135	106	78	37	4	0
90th	—	13.5	15.0	15.3	16.1	18.0	18.0	18.0	—	—
Median	11.0	11.5	12.6	13.8	15.0	15.6	16.6	18.0	18.0	—
10th	—	9.7	11.2	12.0	12.8	14.5	15.3	15.5	—	—
Testosterone (ng/dL)										
No.	4	66	104	76	69	59	32	5	0	0
90th	—	160	336	430	517	575	787	—	—	—
Median	36	36	86	148	259	400	481	569	—	—
10th	—	13	21	38	44	110	327	—	—	—
DHEAS (ng/mL)										
No.	0	4	67	43	32	31	30	6	0	0
90th	—	—	2,320	3,160	3,200	3,560	5,000	—	—	—
Median	—	464	1,160	1,480	1,650	1,840	1,940	2,270	—	—
10th	—	—	420	720	560	1,040	900	—	—	—

Values are number of measurements and percentiles; only the median is shown if *n* ≤10.

**Table 4:** Mean ± SE anthropometric and pubertal measures by maternal *p,p*′-DDE in specific age ranges (years).

*p,p*′-DDE (μg/g lipid)	< 14	14–16.9	17–20
Height (cm)			
< 3	151 ± 1.8 (28)	168 ± 1.9 (26)	174 ± 1.5 (25)
3–5.9	154 ± 0.9 (76)	168 ± 0.9 (86)	176 ± 0.8 (67)
6–8.9	155 ± 1.2 (50)	170 ± 1.3 (50)	176 ± 1.0 (51)
9–11.9	157 ± 2.0 (27)	173 ± 1.7 (24)	177 ± 1.5 (24)
≥ 12	152 ± 1.9 (18)	168 ± 2.1 (21)	173 ± 1.4 (19)
Height ratio (%)			
< 3	50.8 ± 0.3 (28)	51.1 ± 0.4 (26)	51.2 ± 0.3 (25)
3–5.9	50.8 ± 0.1 (76)	50.6 ± 0.2 (84)	51.0 ± 0.2 (66)
6–8.9	50.7 ± 0.2 (50)	50.3 ± 0.2 (50)	50.7 ± 0.2 (51)
9–11.9	50.2 ± 0.3 (27)	50.3 ± 0.3 (24)	50.3 ± 0.3 (24)
≥ 12	50.7 ± 0.3 (18)	50.2 ± 0.3 (21)	50.9 ± 0.4 (18)
BMI (kg/m^2^)			
< 3	17.9 ± 0.5 (28)	21.6 ± 1.0 (26)	21.5 ± 0.6 (25)
3–5.9	20.0 ± 0.5 (76)	20.8 ± 0.5 (85)	22.9 ± 0.4 (67)
6–8.9	18.9 ± 0.4 (50)	20.4 ± 0.5 (50)	22.6 ± 0.4 (51)
9–11.9	18.3 ± 0.5 (27)	21.3 ± 1.0 (24)	22.3 ± 0.9 (24)
≥ 12	18.2 ± 0.5 (18)	19.7 ± 0.6 (21)	21.2 ± 0.6 (19)
Triceps skinfold thickness (mm)			
< 3	8.5 ± 0.6 (28)	10.3 ± 1.5 (26)	7.5 ± 0.6 (25)
3–5.9	11.0 ± 0.8 (76)	8.4 ± 0.5 (86)	8.9 ± 0.6 (67)
6–8.9	9.4 ± 0.7 (50)	8.5 ± 0.7 (50)	8.7 ± 0.6 (51)
9–11.9	8.9 ± 0.9 (27)	9.1 ± 1.5 (24)	8.6 ± 1.4 (24)
≥ 12	8.1 ± 0.7 (18)	8.2 ± 0.8 (21)	7.2 ± 0.8 (19)
Central adiposity (%)			
< 3	46.2 ± 0.8 (28)	49.1 ± 1.0 (26)	56.8 ± 0.9 (25)
3–5.9	47.6 ± 0.6 (75)	51.9 ± 0.6 (86)	56.1 ± 0.6 (67)
6–8.9	47.7 ± 0.7 (50)	51.2 ± 0.7 (50)	55.3 ± 0.8 (51)
9–11.9	48.0 ± 0.7 (27)	50.8 ± 1.0 (24)	58.1 ± 0.9 (24)
≥ 12	46.5 ± 1.1 (18)	50.7 ± 1.0 (21)	57.2 ± 1.3 (19)
Skeletal age (years)			
< 3	12.4 ± 0.3 (28)	15.8 ± 0.3 (24)	
3–5.9	13.1 ± 0.1 (72)	15.4 ± 0.1 (73)	
6–8.9	13.0 ± 0.2 (50)	15.4 ± 0.2 (43)	
9–11.9	13.1 ± 0.3 (27)	15.9 ± 0.2 (22)	
≥ 12	13.0 ± 0.3 (18)	15.6 ± 0.3 (21)	
Testosterone (ng/dL)			
< 3	124 ± 26 (26)	376 ± 33 (18)	
3–5.9	136 ± 17 (63)	321 ± 28 (43)	
6–8.9	172 ± 25 (42)	346 ± 46 (25)	
9–11.9	111 ± 19 (24)	491 ± 103 (11)	
≥ 12	142 ± 41 (13)	333 ± 52 (15)	
DHEAS (ng/mL)			
< 3	1,355 ± 184 (20)	2,248 ± 274 (16)	
3–5.9	1,431 ± 142 (40)	1,901 ± 168 (35)	
6–8.9	1,428 ± 186 (28)	1,911 ± 218 (19)	
9–11.9	1,236 ± 186 (18)	2,352 ± 476 (10)	
≥ 12	1,240 ± 180 (8)	2,186 ± 492 (13)	

For each boy, all available measurements in the specified age range are averaged. Values are mean ± SE of these averages (no. of boys). Skeletal age and hormone concentrations are not shown in the upper age range because they were available for few boys.

**Table 5:** Regression of anthropometric and pubertal measures on maternal *p,p*′-DDE and other predictors.

Predictor, category/units	Height (cm)	Height ratio (%)	BMI (kg/m^2^)	Triceps (log mm)	Central adiposity (%)	Skeletal age (years)	Testosterone (log ng/dL)	DHEAS (ng/mL)
Maternal *p,p*′-DDE (μg/g lipid)								
< 3	0 (ref)	0 (ref)	0 (ref)	0 (ref)	0 (ref)	0 (ref)	0 (ref)	0 (ref)
3–5.9	1.1 ± 1.3	0.1 ± 0.2	0.9 ± 0.7	0.06 ± 0.08	1.1 ± 0.9	0.4 ± 0.2	0.0 ± 0.2	−158 ± 217
6–8.9	1.0 ± 1.5	0.1 ± 0.2	0.2 ± 0.8	0.00 ± 0.09	0.5 ± 1.0	0.3 ± 0.3	0.1 ± 0.2	−140 ± 252
9–11.9	2.2 ± 1.7	0.0 ± 0.3	0.6 ± 0.9	0.00 ± 0.10	1.7 ± 1.2	0.5 ± 0.3	0.0 ± 0.2	−109 ± 281
≥ 12	0.4 ± 1.8	0.0 ± 0.3	−0.4 ± 0.9	−0.01 ± 0.11	0.6 ± 1.2	0.2 ± 0.3	0.1 ± 0.2	−148 ± 307
Maternal height per 10 cm	4.4 ± 0.7**	−0.5 ± 0.1**	−0.2 ± 0.4	0.05 ± 0.04	0.0 ± 0.5	−0.1 ± 0.1	−0.1 ± 0.1	−32 ± 114
Maternal BMI per 10 kg/m^2^	2.0 ± 1.0*	0.0 ± 0.2	2.2 ± 0.5**	0.25 ± 0.06**	−0.3 ± 0.7	0.2 ± 0.2	0.2 ± 0.1	280 ± 179
Race								
White	−0.9 ± 1.3	2.1 ± 0.2	0.4 ± 0.7	0.15 ± 0.08	−2.8 ± 0.9	0.0 ± 0.2	0.0 ± 0.2	−110 ± 227
African American	0 (ref)	0 (ref)**	0 (ref)	0 (ref)*	0 (ref)**	0 (ref)	0 (ref)	0 (ref)
No. of older siblings								
0	0 (ref)**	0 (ref)*	0 (ref)	0 (ref)**	0 (ref)	0 (ref)	0 (ref)	0 (ref)
1	−1.1 ± 1.3	0.0 ± 0.2	−0.6 ± 0.7	−0.11 ± 0.08	1.4 ± 0.9	−0.3 ± 0.2	−0.2 ± 0.2	−320 ± 235
≥ 2	−3.2 ± 1.4	0.4 ± 0.2	−0.9 ± 0.7	−0.21 ± 0.08	1.7 ± 0.9	−0.5 ± 0.2	−0.2 ± 0.2	−522 ± 245
Maternal smoking in pregnancy								
No	0 (ref)	0 (ref)	0 (ref)	0 (ref)	0 (ref)	0 (ref)	0 (ref)	0 (ref)
Yes	−0.2 ± 0.8	−0.2 ± 0.1	0.3 ± 0.5	0.02 ± 0.05	0.7 ± 0.6	0.0 ± 0.1	0.0 ± 0.1	60 ± 152
Breast-fed								
No	0.4 ± 1.7	−0.3 ± 0.3	0.5 ± 0.9	0.12 ± 0.10	−0.7 ± 1.2	−0.1 ± 0.3	0.1 ± 0.2	−247 ± 285
Yes	0 (ref)	0 (ref)	0 (ref)	0 (ref)	0 (ref)	0 (ref)	0 (ref)	0 (ref)
Family SEI								
≥ 5.1	2.4 ± 1.4	0.4 ± 0.2	1.1 ± 0.7	0.12 ± 0.08	−0.3 ± 0.9	0.4 ± 0.2	0.4 ± 0.2	207 ± 238
2.6–5.0	2.5 ± 1.2	0.1 ± 0.2	0.7 ± 0.6	0.04 ± 0.07	0.0 ± 0.8	0.4 ± 0.2	0.2 ± 0.2	184 ± 204
0–2.5	0 (ref)*	0 (ref)	0 (ref)	0 (ref)	0 (ref)	0 (ref)	0 (ref)*	0 (ref)
Maternal age at enrollment (years)								
13–19	−0.9 ± 1.8	−0.1 ± 0.3	−0.7 ± 1.0	−0.20 ± 0.11	1.7 ± 1.2	−0.4 ± 0.3	0.2 ± 0.2	−340 ± 318
20–24	−0.6 ± 1.3	−0.3 ± 0.2	−0.5 ± 0.7	−0.08 ± 0.08	0.4 ± 0.9	−0.2 ± 0.2	−0.1 ± 0.2	−122 ± 237
25–29	0.2 ± 1.4	−0.4 ± 0.2	0.2 ± 0.7	−0.05 ± 0.08	0.5 ± 1.0	0.0 ± 0.2	0.2 ± 0.2	−252 ± 243
≥ 30	0 (ref)	0 (ref)	0 (ref)	0 (ref)	0 (ref)	0 (ref)	0 (ref)	0 (ref)
Maternal age at menarche (years)								
8–11	0 (ref)	0 (ref)*	0 (ref)	0 (ref)	0 (ref)	0 (ref)	0 (ref)*	0 (ref)
12	−0.6 ± 1.2	−0.5 ± 0.2	0.0 ± 0.6	0.03 ± 0.07	−1.2 ± 0.8	−0.3 ± 0.2	−0.5 ± 0.2	−77 ± 212
13	0.3 ± 1.3	−0.4 ± 0.2	−0.4 ± 0.7	0.00 ± 0.08	−0.9 ± 0.9	−0.2 ± 0.2	−0.3 ± 0.2	47 ± 228
≥ 14	1.0 ± 1.3	−0.4 ± 0.2	−0.3 ± 0.7	0.00 ± 0.08	−1.3 ± 0.9	−0.4 ± 0.2	−0.3 ± 0.2	−52 ± 223

ref, reference. Values are regression coefficients ± SE, adjusted for all others shown and also for age of boy at measurement and correlation among multiple measures of the same boy.

* *p* < 0.10,

** *p* < 0.05, for tests of whether discrete groups differ or continuous slope is nonzero.
